# Role of the Annexin A protein family in liver diseases: insights and therapeutic opportunities

**DOI:** 10.3389/fphar.2025.1569927

**Published:** 2025-07-11

**Authors:** Mingyang Feng, Yong He, Hui Wang

**Affiliations:** ^1^ Department of Infectious Diseases, Ruijin Hospital, Shanghai Jiao Tong University School of Medicine, Shanghai, China; ^2^ Shanghai Institute of Materia Medica, Chinese Academy of Sciences, Shanghai, China

**Keywords:** annexins, ANXA2, liver disease, MAFLD, HCC

## Abstract

The Annexin (ANXA) protein family, which is ubiquitously expressed across various tissues, encodes versatile calcium (Ca^2+^)- and phospholipid-binding proteins that play crucial roles in modulating inflammation and cell signaling pathways. This family significantly influences several essential cellular processes, including cell adhesion, proliferation, migration, differentiation, and apoptosis. ANXAs are integral to physiological regulation and the pathological states associated with liver diseases. Dysregulated expression of ANXAs has been linked to a spectrum of liver conditions, including metabolic dysfunction, hepatocyte damage, fibrosis, and tumor formation. In this review, we outline recent advancements in understanding the roles of ANXAs in liver diseases. Further investigation into the roles of ANXAs in the liver could enhance our understanding of the mechanisms underlying liver diseases and may identify biomarkers and therapeutic targets for liver diseases in the future.

## 1 Introduction

Liver diseases encompass pathological alterations in the liver induced by a diverse array of internal and external pathogenic factors, thereby disrupting normal physiological functions. Liver diseases can be triggered by various causes, including viral infections, metabolic disorders, drug-induced effects, excessive alcohol consumption, and autoimmune aberrations (Asrani et al., 2019). Depending on their etiology and pathogenesis, liver diseases are predominantly classified into viral hepatitis, metabolic dysfunction-associated fatty liver disease (MAFLD), drug-induced liver injury (DILI), alcoholic liver disease (ALD), autoimmune hepatitis (AIH), primary biliary cholangitis (PBC), primary sclerosing cholangitis (PSC), cirrhosis, and hepatocellular carcinoma (HCC) (Xiao et al., 2019). In recent decades, liver diseases have become one of the leading causes of death and illness worldwide (Xiao et al., 2019), resulting in over two million fatalities annually and constituting 4% of global deaths (Devarbhavi et al., 2023). Understanding the mechanisms underlying liver diseases and advancing targeted treatment strategies are crucial for improving clinical outcomes.

Annexins (ANXAs) constitute a family of calcium-regulated, membrane-associated proteins (Gerke et al., 2024). The first ANXA proteins were identified through biochemical methods that capitalized on their unique membrane-binding properties. Over the past few decades, extensive research has revealed that ANXAs are involved in a wide array of physiological and pathological processes, profoundly influencing diverse cellular functions (Li Y. Z. et al., 2022; Xi et al., 2020). A complex relationship between ANXA expression and various liver diseases has also been established. In recent years, there has been increasing evidence of a strong association between ANXAs and various liver diseases. Notably, ANXAs play an important role in the pathogenesis of liver diseases by regulating liver metabolism, inflammation, fibrosis, immune cell function, regeneration, and tumor development (Wu et al., 2024). In addition, certain types of ANXAs can be detected in the extracellular environment, such as in the blood, where they correlate with disease processes, suggesting their potential as biomarkers. In this review, we evaluate numerous studies on the role of ANXAs in liver disease, explore potential therapeutic strategies targeting ANXAs, and provide an outlook for future research.

## 2 Functions of annexins

ANXAs were originally discovered in the late 1970s to early 1980s. The term “Annexin” is derived from the Latin word anectere, meaning “to bind or connect,” reflecting their capacity to bind phospholipids in a calcium-dependent manner (Vedeler et al., 2025). The 12 vertebrate annexins, designated ANXA1 through ANXA11 and ANXA13, belong to the Annexin A subfamily (White et al., 2024). All ANXA proteins contain conserved core domains of approximately 70 amino acids and are characterized by variable N-terminal regions and calcium-binding sites that facilitate membrane interaction (Hakami Zanjani et al., 2024). These proteins also contain binding sites for cytoplasmic protein ligands and can be targeted to cellular membranes through their core-mediated phospholipid-binding activity (Hakami Zanjani et al., 2024).

ANXAs are widely expressed across various tissues (Supplementary Table S1) and are implicated in numerous cellular processes, including membrane scaffolding, ion channel regulation, vesicular trafficking, membrane repair, cell signaling, proliferation, differentiation, apoptosis, and migration (Gerke et al., 2024; Gerke et al., 2005; Gerke and Moss, 2002). Although traditionally regarded as intracellular proteins, some ANXAs have also been detected in the extracellular space. However, the mechanisms governing their extracellular secretion and their functions outside the cell remain poorly understood.

## 3 Annexin A protein family in liver diseases

The ANXAs play an important role in liver diseases such as HCC, MAFLD, liver fibrosis, and viral hepatitis (Table 1). Importantly, ANXAs are expected to serve as biomarkers and potential therapeutic targets for liver diseases, thus advancing the diagnosis and treatment of liver diseases in the future.

**TABLE 1:** Role and mechanism of ANXAs in liver diseases.

Diseases	ANXAs members	Function	Molecular mechanisms
HBV	ANXA2	Promotion of HBV infection	Receptor for β2GP I; Negative regulation JAK/STAT/BST2
		Promotion of MTCT	S100A10/ANXA2 compound
	ANXA5	Promotion of HBV infection	Receptor for HBV
		Inhibit HBV replication; reduce HBsAg secretion	Enhanced endocytosis/exocytosis of viral particles
HCV	ANXA2	Promotion of HCV infection	Contributes to the assembly of HCV
	ANXA3	Promotion of HCV infection	Contributes to the interaction of the viral envelope protein E2 and ApoE to assist HCV particle formation
	ANXA5	Promotion of HCV infection	ANXA5 downregulates PKCα and PKCη and thereby reduces OCLN phosphorylation
MAFLD/MASH	ANXA1	Reduces inflammation and fibrosis	Reduced macrophage M1 polarization
	ANXA2	Exacerbates lipid accumulation and fibrosis	Activates Caspase-1; Disruption of AMPK/mTOR-mediated lipophagy
	ANXA5	Improvement of lipid accumulation, reduction of inflammatory cell infiltration, inhibition of fibrosis	Promotes macrophage M1 to M2 polarization
	ANXA9	Unclear	Unclear
PSC	ANXA1	Unclear	Unclear
PBC	ANXA2	Promotes cholestasis	Regulates PKC activity
ALD	ANXA2	Unclear	Unclear
Liver fibrosis	ANXA1	Reduce liver fibrosis	ANXA1 targets FPR to regulate macrophage function and thereby inhibit the Wnt/β-catenin pathway in HSC
	ANXA2	Promotes liver fibrosis	Promotion of STAT3 phosphorylation upregulates α-SMA expression
	ANXA3	Unclear	Unclear
	ANXA4	Unclear	Unclear
HCC	ANXA1	Promote tumor proliferation and migration	Reduced M1/M2 macrophage ratio and decreased T-cell activation
	ANXA2	Promote tumor migration and invasion	Regulates the transport of CD147-containing vesicles; Remodeling cellular structures
		EMT	Activates Wnt/β-catenin signaling by binding to lncRNA-MUF
		Promoting convergence and transfer	Interacts with ELMO1
	ANXA3	Promote tumor cell survival	Inhibition of PKCδ/p38-mediated apoptosis with simultaneous activation of autophagy
		Enhanced tumor stem-like features	Downregulation of the JNK signaling pathway; Adjusts the HIF1A/Notch signal
	ANXA4	Tumor suppression	Unclear
	ANXA5	Promote tumor metastasis and invasion	Through integrin and MEK/ERK pathways
	ANXA7	Promoting tumor metastasis	Interaction with SRI
		Inhibition of tumor metastasis	Unclear
	ANXA10	Tumor suppression	Related to p53 mutation
Liver regeneration	ANXA6	Promote liver regeneration	Promoting membrane localization and functional recovery of SNAT4

### 3.1 Viral hepatitis B

**FIGURE 1:**
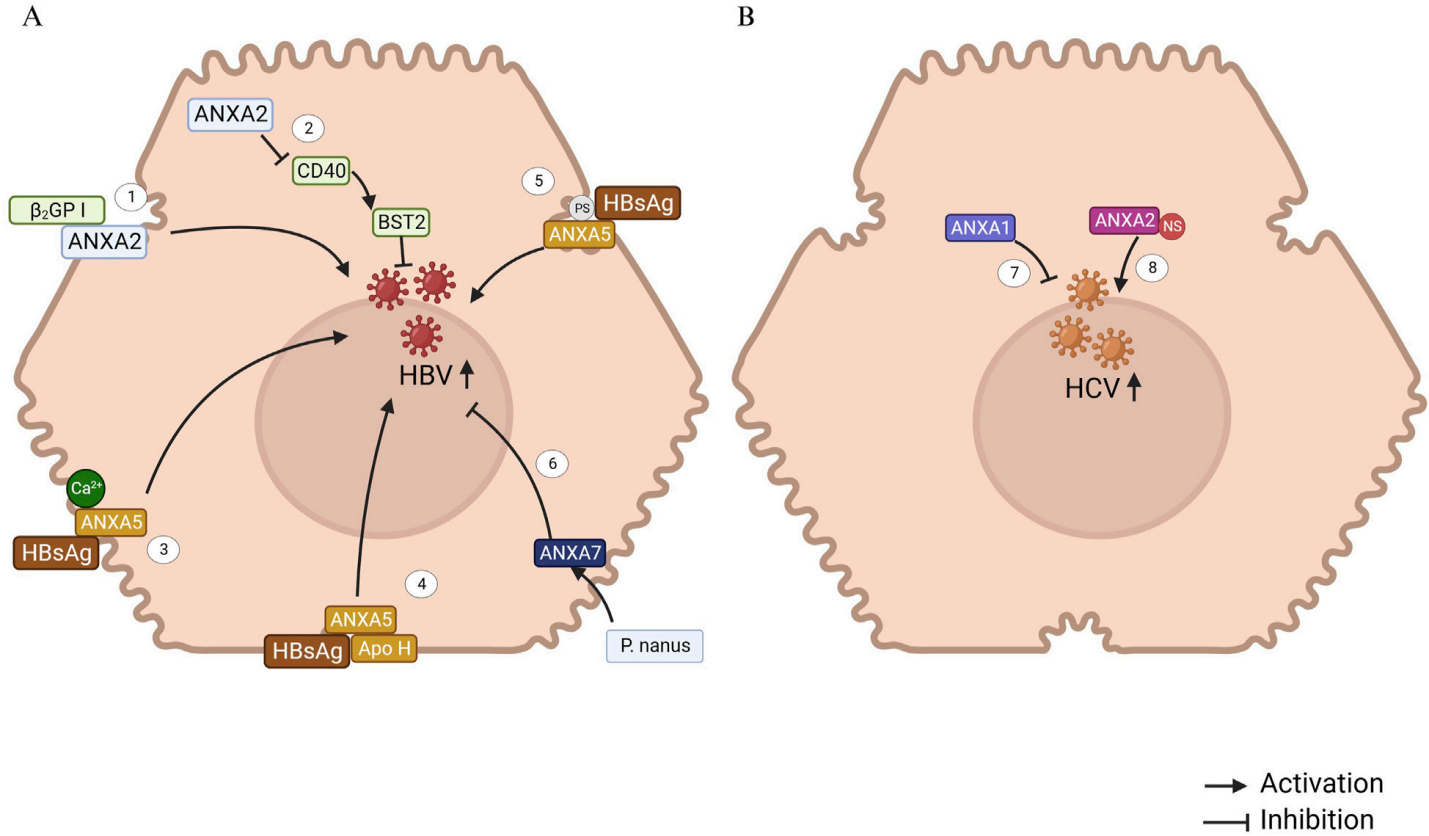
ANXAs involved in Viral hepatitis B and C. **(A)** 1. ANXA2, located on the hepatocyte membrane, acts as the receptor for β(2)GP I, potentially facilitating the interaction between HBV and infected hepatocytes (Gao et al., 2007). 2. ANXA2, a regulatory protein, downregulates CD40 expression, which subsequently reduces the expression of the IFN-stimulated gene BST2, thereby promoting HBV replication and transcription (Chen et al., 2023). 3. Hepatic plasma membrane protein ANXA5 binds specifically to HBsAg in a Ca^2+^-dependent manner, indicating its crucial role in the progression of HBV infection (Hertogs et al., 1993). 4. ANXA5, along with apolipoprotein H (Apo H), binds to the hepatitis B surface antigen (HBsAg). It is hypothesized that these proteins specifically interact with the HBsAg S protein, playing a significant role in the initiation of HBV infection (Neurath and Strick, 1994). 5. Phosphatidylserine, a phospholipid component of the HBV envelope, plays a crucial role in the binding of ANXA5 to the HBV envelope and is essential for HBV infection *in vitro* (De Meyer et al., 1999). 6. An ethanolic extract of *Phyllanthus nanus* (*P. nanus*) demonstrated strong antiviral activity against HBV. ANXA7 may significantly contribute to the therapeutic effects of the active components in *P. nanus* (Lam et al., 2006). **(B)** 7. ANXA1 inhibits HCV RNA replication but does not affect the viral entry process into hepatocytes (Hiramoto et al., 2015). 8. HCV RNA replication in hepatocytes occurs through the formation of a replication complex, composed of both viral nonstructural (NS) proteins and host cell proteins. ANXA2 is involved in this complex, where it interacts with the NS proteins (Saxena et al., 2012).

Hepatitis B virus (HBV) infection remains to be a significant global health issue, with chronic infection potentially progressing to cirrhosis or even HCC (Feng M. et al., 2022). β2-glycoprotein I (β2GP I), a plasma glycoprotein, has been shown to bind to recombinant hepatitis B surface antigen (rHBsAg), suggesting a role in facilitating HBV entry into hepatocytes. A recent study found that ANXA2, located on the membrane of the SMMC-7721 HCC cell line, served as a receptor for β2GP I, suggesting that ANXA2 may play a bridging role in HBV infection of hepatocytes (Figure 1A) (Gao et al., 2007). Furthermore, upregulation of ANXA2 has been observed in HBV-replicating Hep RG cells (Narayan et al., 2009) and in Hep G2 cells transfected with HBV X protein (HBx) (Feng et al., 2010). Mechanistically, HBV infection increases the expression of ETS variant 4 (ETV4), which in turn enhances ANXA2 expression at the transcriptional level through binding to the ANXA2 promoter (Sun and Zhang, 2021). Moreover, CD40 mediates anti-HBV effects by upregulating the Janus kinase (JAK)/Signal Transducer and Activator of Transcription (STAT)/bone marrow stromal cell antigen 2 (BST2) axis, which is negatively regulated by ANXA2 (Chen et al., 2023).

Interestingly, ANXA2 expression is downregulated in primary duck liver cells (PDHs) infected with duck hepatitis B virus (DHBV) (Zhao et al., 2010). The HepG2.2.15 cell line, a human HCC line stably transfected with the HBV genome, exhibits sustained HBV expression. In contrast, the parental HepG2 cell line lacks integrated HBV DNA and does not support viral replication. Notably, ANXA2 expression is significantly downregulated in HepG2.2.15 cells compared with HBV-negative HepG2 cells, suggesting a potential functional link between ANXA2 and HBV infection or replication (Niu et al., 2009). ANXA2 expression exhibits divergent patterns during HBV infection, possibly due to variations in species or cell models. Future investigations should standardized experimental conditions to definitively characterize the mechanistic role of ANXA2 in HBV pathogenesis.

Mother-to-child transmission (MTCT) is a primary route for chronic HBV infection. In intrauterine HBV infection and MTCT, a portion of the virus utilizes the autophagic protein secretion pathway, translocating across the trophoblastic layer via exocytosis facilitated by the S100 Calcium Binding Protein A10 (S100A10)/ANXA2 complex and polyvesicular bodies (Bai et al., 2022). This study identifies a potential therapeutic target for disrupting the mechanisms underlying HBV intrauterine transmission and vertical mother-to-child infection.

ANXA5, a protein present in fetal tissue, may also serve as an HBV receptor across various tissues. Elevated ANXA5 levels in the liver might contribute to the organ’s increased susceptibility to HBV infection (Ye et al., 2006). The binding of viral envelope proteins to specific receptors on hepatocytes is a crucial step in HBV infection. A previous study has demonstrated that ANXA5, a human liver plasma membrane protein, specifically binds to small hepatitis B surface antigen (HBsAg) in a calcium-dependent manner, highlighting its potential role in HBV infection (Hertogs et al., 1993). ANXA5 plays a key role in the early stages of HBV infection, and further research suggests that species-specific susceptibility to HBV infection and replication in hepatocytes is linked to ANXA5 expression (Gong et al., 1999). In rat hepatocyte primary cultures, ANXA5 promotes HBV entry, facilitating successful infection, while in human hepatocyte primary cultures, ANXA5 does not prevent HBV infection (De Meyer et al., 2000). Additionally, ANXA5 and apolipoprotein H have been shown to bind to HBsAg, specifically interacting with the HBsAg S protein, which is essential for initiating HBV infection (Neurath and Strick, 1994). The binding of phosphatidylserine and non-phospholipid components of the HBV envelope to ANXA5 also contributes to the infection process (De Meyer et al., 1999). Collectively, the evidence indicates that ANXA5 plays a crucial role in HBV infection through facilitating viral entry into hepatocytes, highlighting its potential as a therapeutic target for anti-HBV drug development.

ANXA7 plays a critical role in modulating HBsAg release during HBV infection. It has been demonstrated that the ethanolic extract of *Phyllanthus nanus* upregulates ANXA7 expression in HBV-infected hepatoma cells, which correlates with suppressed HBV replication and reduced HBsAg secretion. ANXA7 localizes near secretory vesicles and may inhibit HBV by enhancing endocytosis/exocytosis of viral particles or interfering with HBsAg release. Functional studies have confirmed that ANXA7 overexpression in HBV-integrated Alexander cells markedly reduces extracellular HBsAg levels (Lam et al., 2006). These findings position ANXA7 as a promising molecular target for novel HBV therapeutics aimed at blocking viral propagation by hijacking vesicular trafficking pathways.

### 3.2 Viral hepatitis C

Chronic infection with hepatitis C virus (HCV) often progresses to chronic hepatitis, which has a high likelihood of advancing to cirrhosis and HCC (Hajarizadeh et al., 2023). ANXA1 plays a role in inhibiting HCV RNA replication, although it does not affect the initial viral entry into human hepatocytes (Hiramoto et al., 2015).

HCV RNA replication complex (RC) is formed by viral nonstructural (NS) proteins and host cell proteins, enabling the replication of the viral RNA genome associated with the cell membrane (Lai et al., 2008). The enzymatic activity of these proteins plays a crucial role in the HCV replication process. Previous studies have shown that ANXA2 interacts with NS3/NS4A (Lai et al., 2008) and helps recruit HCV NS proteins, concentrating them to form replication complexes (Figure 1B) (Saxena et al., 2012). Although silencing ANXA2 expression does not affect viral RNA replication, it results in a significant reduction in both extracellular and intracellular viral titers. This suggests that ANXA2 likely contributes to HCV assembly rather than genome replication or the release of viral particles. Colocalization studies of separately expressed HCV NS proteins have indicated that NS5A may specifically recruit ANXA2 through indirect mechanisms (Backes et al., 2010).

Knocking down ANXA3 does not affect HCV RNA replication, but it does significantly disrupt the production of viral particles. Mechanistically, ANXA3 plays a critical role in the interaction between the viral envelope protein E2 and apolipoprotein E (ApoE), as well as in the transport (but not the lipidogenesis) of ApoE in HCV-infected cells (Rösch et al., 2016). Therefore, ANXA3 may serve as a co-factor for HCV particle production.

The disruption of occludin (OCLN) distribution facilitates HCV infection. Normal distribution of OCLN is regulated by phosphorylation. Knockout of ANXA5 results in decreased phosphorylation of OCLN, thereby leading to its disrupted distribution and promoting HCV infection. Protein kinase C (PKC) subtypes, such as PKCα and PKCη, play a crucial role in regulating ANXA5-mediated OCLN phosphorylation and distribution, which in turn helps limit HCV infection. HCV infection downregulates the expression of PKCα and PKCη, thereby reducing OCLN phosphorylation (Abe et al., 2023). Collectively, the data indicate that ANXA5 mimics could function as effective HCV entry inhibitors.

### 3.3 Metabolic dysfunction-associated fatty liver disease

MAFLD has emerged as the most prevalent chronic liver disorder worldwide, affecting an estimated 38% of the global population (Wong et al., 2023). This disease encompasses a broad spectrum of severity, ranging from simple steatosis to metabolic dysfunction-associated steatohepatitis (MASH), cirrhosis, and HCC (Munk Lauridsen et al., 2025). MAFLD is intrinsically linked to metabolic comorbidities, particularly obesity, insulin resistance, type 2 diabetes mellitus, and atherogenic dyslipidemia (Mejía-Guzmán et al., 2025). Compared with the general population, MAFLD patients exhibit increased risks of liver-related, kidney-related, cardiovascular, and all-cause mortality (Cao et al., 2021; Mann et al., 2020). Emerging mechanistic evidence highlights ANXAs as critical regulators in MAFLD pathogenesis, modulating key processes such as lipid metabolism, inflammatory signaling, and fibrosis progression.

Exogenous treatment with ANXA1 has demonstrated efficacy in counteracting the progression of MASH, primarily through its anti-inflammatory and anti-fibrotic properties, although its impact on hepatic steatosis appears to be limited. In preclinical models, MASH was induced in mice via feeding of a methionine-choline deficient (MCD) diet or a Western diet (WD). Once MASH was established, the animals received daily intraperitoneal (IP) injections of human recombinant ANXA1 (hrANXA1; 1 μg) or saline for 4–6 weeks. Across both experimental paradigms, hrANXA1 treatment significantly alleviated liver injury and reduced inflammatory cell infiltration, without influencing the degree of steatosis (Gadipudi et al., 2022). Mechanistically, macrophage-derived ANXA1 ameliorates hepatic inflammation and fibrosis by reducing macrophage M1 polarization during MASH progression (Locatelli et al., 2014). However, the molecular mechanisms governing ANXA1-mediated regulation of macrophage polarization and function remain elusive. This knowledge gap represents a crucial area that merits comprehensive investigation in future research endeavors.

ANXA2 is significantly upregulated in both MAFLD patients and high-fat diet (HFD)-fed mouse models, where it exacerbates MAFLD-associated lipid accumulation and fibrosis (Sobolewski et al., 2020), and ANXA2 expression exhibits a positive correlation with the progression of MAFLD-associated hepatocyte pyroptosis and fibrosis (Feng Y. et al., 2022). Bioinformatics analysis showed that ANXA2 could act as a core gene driving MASH progression (Arendt et al., 2019; Li X. et al., 2022; Qin et al., 2023; Chen et al., 2024; Fan et al., 2024). Mechanistic studies have shown that ANXA2 activates Caspase-1-mediated MASH hepatocyte pyroptosis and fibrosis (Feng Y. et al., 2022). Another study showed that ANXA2 promotes lipid accumulation and liver injury by disrupting AMP-activated protein kinase (AMPK)/mechanistic target of rapamycin (mTOR)-mediated lipophagy (Wu et al., 2024). In addition, increased ANXA2 expression in hepatocytes promotes MASH-associated hepatic fibrosis by increasing the expression of osteopontin (Wang et al., 2022). Thus, future studies may focus on investigating the potential of ANXA2 as a pathological predictor for MAFLD and a promising therapeutic target.

**FIGURE 2:**
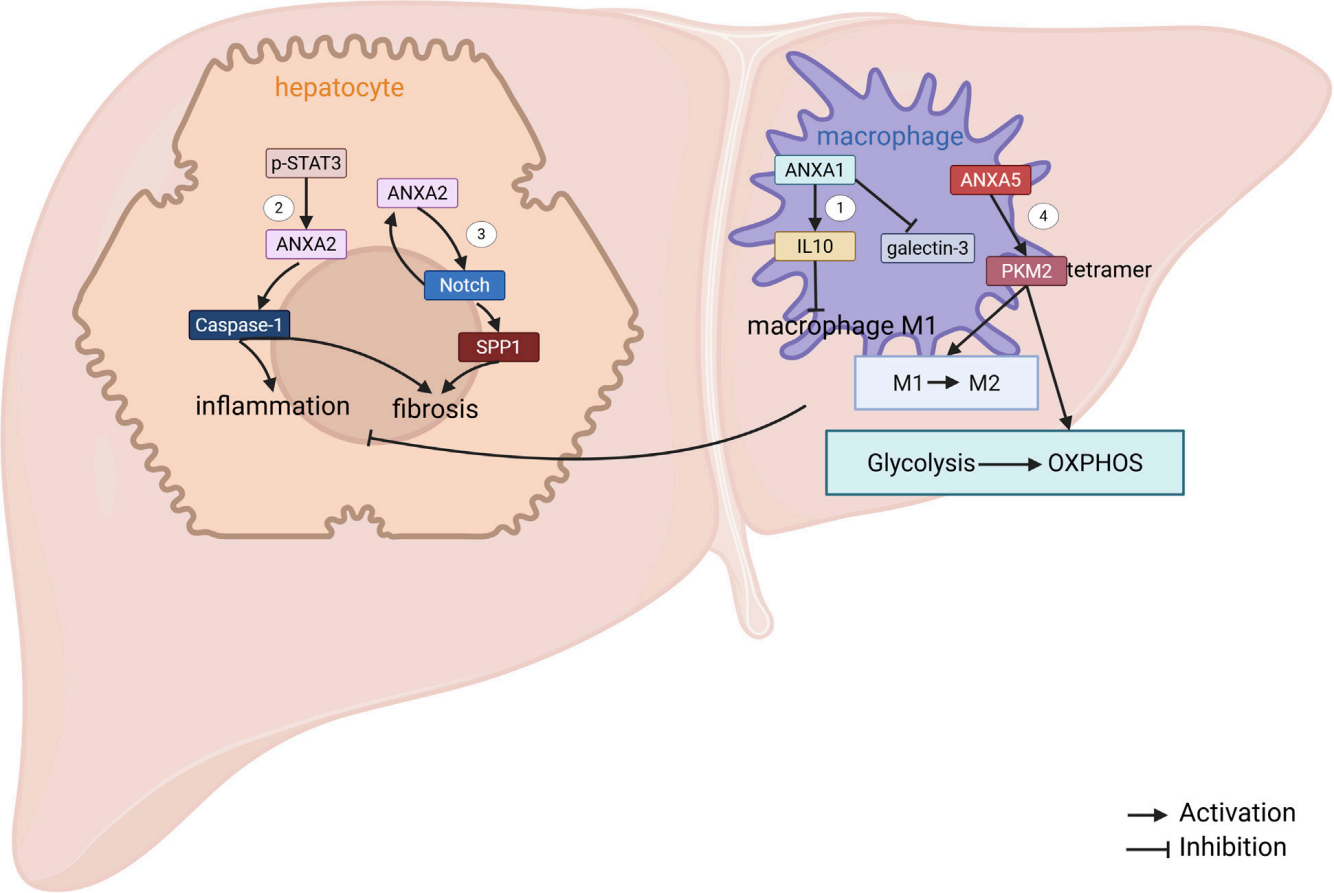
ANXAs involved in MAFLD. 1. In NASH mice, macrophage-derived ANXA1 reduces M1 polarization by promoting interleukin-10 (IL-10) production. Additionally, ANXA1 downregulates the expression of galactoglucan-3 (Locatelli et al., 2014). 2. Phosphorylated STAT3 (p-STAT3) enhances the transcriptional expression of ANXA2, which in turn triggers Caspase-1-mediated pyroptosis in NASH hepatocytes, contributing to liver fibrosis. 3. In hepatocytes, the ANXA2-Notch positive regulatory loop activates hepatic stellate cells (HSCs) by upregulating SPP1 expression, thus promoting liver fibrosis in NASH (Wang et al., 2022). 4. ANXA5 directly interacts with pyruvate kinase M2 (PKM2), which leads to a shift in metabolism from glycolysis to oxidative phosphorylation (OXPHOS), induces phenotypic changes in hepatic macrophages, and alleviates NASH (Xu et al., 2020).

ANXA5 attenuated MASH-associated hepatic lipid accumulation, reduced inflammatory cell infiltration, and suppressed fibrosis. Mechanistically, in hepatic macrophages, ANXA5 directly binds to pyruvate kinase M2 (PKM2) at the ASP101, LEU104, and ARG106 residues. This interaction facilitates the assembly of active PKM2 tetramers while inhibiting PKM2 Y105 phosphorylation. By enhancing PKM2’s pyruvate kinase activity, ANXA5 drives metabolic reprogramming in M1 macrophages, shifting their energy metabolism from glycolysis to oxidative phosphorylation (OXPHOS). Consequently, ANXA5 promotes a phenotypic switch of hepatic macrophages from pro-inflammatory M1 to anti-inflammatory M2 polarization, thereby mitigating MASH progression (Figure 2) (Xu et al., 2020). Notably, intravenous administration of ANXA5 in HFD-induced MASH mice alleviates hepatic lipotoxicity, inflammation, and fibrosis, underscoring its therapeutic potential for clinical translation.

Furthermore, bioinformatics analysis identified ANXA9 as a key driver gene in MAFLD pathogenesis, indicating its potential utility as a diagnostic biomarker and therapeutic target (Song et al., 2023a).

### 3.4 Autoimmune liver disease

PSC is a chronic cholestatic liver disease characterized by bile duct stenosis due to inflammation and fibrosis of the intrahepatic and extrahepatic bile ducts, which can ultimately progress to cirrhosis and liver failure (Manns et al., 2025). The pathogenesis of PSC has not been fully elucidated yet, and it may be due to a combination of factors such as genetics, environment, immunity, bile acid metabolism disorders, and dysfunction of intestinal flora (Tan et al., 2023).

ANXA1 expression was significantly upregulated in liver tissues and bile of PSC patients. T cell infiltration, which is thought to play a crucial role in PSC. Bioinformatics analysis of one study showed that ANXA1 is a key gene associated with high risk and infiltration of immune cells, especially T cells, in PSC (Zhang J. et al., 2023). Staining of hepatic tissues for ANXA1 showed that it was significantly upregulated around the hepatic tissues of the portal vein in patients with PSC. Although ANXA1 was not expressed predominantly on T cells, the areas of high ANXA1 expression were also accompanied by a greater number of CD3^+^ T cells infiltrating (Zhang J. et al., 2023). In another study, bile proteomic analysis showed that ANXA1 was significantly upregulated in the bile of patients with PSC, and immunostaining of hepatic tissue for ANXA1 showed that, in addition to its expression in cholangiocytes and vascular endothelial cells, it also highly expressed in inflammatory cells infiltrating the peripheral bile ducts (Kan et al., 2023). Both studies only demonstrated the expression of ANXA1 in patients with PSC and did not investigate the biological role of ANXA1 in PSC. Previous studies have shown that ANXA1 belongs to the group of anti-inflammatory proteins (Perretti and D'Acquisto, 2009; Perretti and Dalli, 2023; Gavins and Hickey, 2012), and its anti-inflammatory effects are exerted by inhibiting the release of inflammatory mediators (e.g., prostaglandin E2 and leukotrienes), promoting tissue repair, and enhancing leukocyte migration (Purvis et al., 2019). However, the exact role of ANXA1 in PSC, the specific mechanism of its action, and the mechanism for the increased expression of ANXA1 in bile remain to be elucidated.

PBC is an autoimmune liver disease characterized by progressive destruction of intrahepatic bile ducts leading to cholestasis, cirrhosis and liver failure (Tanaka, 2024). Proteomic analysis of PBC patients showed significant upregulation of ANXA2 expression in cholangiocytes. Preliminary functional analyses suggest that the upregulation of ANXA2 expression in cholangiocytes may promote cholestasis by regulating protein kinase C (PKC) activity to compensate for the impaired anion exchanger (AE) activity in cholangiocytes in PBC, i.e., bicarbonate-rich ductal secretion and bile formation. However, the specific regulatory mechanisms, such as molecule-to-molecule interactions, by which ANXA2 functions in PBC remain to be further explored (Kido et al., 2009).

### 3.5 Alcohol-related liver disease

ALD encompasses a range of hepatic pathologies, including steatosis, hepatitis and cirrhosis, that develop secondary to prolonged alcohol abuse. While the progression of ALD is primarily influenced by the amount and duration of alcohol intake, and it is also shaped by genetic, epigenetic, and environmental factors (Liu S. Y. et al., 2021). It has been demonstrated that ANXA2 expression is significantly elevated in alcohol-induced cell lines, in mouse and baboon models of ALD, and in liver tissues of ALD patients (Seth et al., 2008; Seth et al., 2003; Zhang et al., 2011). Although the relationship between ANXAs and ALD has not been extensively studied, emerging evidence suggests that ANXAs may play a role in the progression of liver disease. Further research is needed to clarify their mechanisms in ALD.

### 3.6 Liver fibrosis

Liver fibrosis is associated with chronic liver injury including viral hepatitis, autoimmune liver disease and MAFLD. As the number of patients affected by virus-related liver disease decreases with the availability of antiviral drugs, the increase in fibrosis in patients with MAFLD has now become one of the most critical issues in the field of hepatology. The progression of liver fibrosis in response to injury involves complex interactions between multiple cell types in the liver, and there is a close link between hepatocellular injury, activation of innate immune cells, and the production of extracellular matrixc (ECM) (Rieder et al., 2025). MAFLD-associated liver fibrosis has been demonstrated in the “Metabolic dysfunction-associated fatty liver disease” chapter.

ANXA1 is upregulated in fibrosis. Functional studies showed that ANXA1 attenuated CCl4-induced hepatic fibrosis in mice, and the mechanism may be that ANXA1 targets the N-formylpeptide receptor (FPR) to regulate macrophage function and thus inhibits Wnt/β-catenin pathway activation in hepatic stellate cell (HSC) (Fan et al., 2023). Thus, the fibrosis inhibitory effect of ANXA1 makes it a potential for future development of drugs for the treatment of liver fibrosis.

**FIGURE 3:**
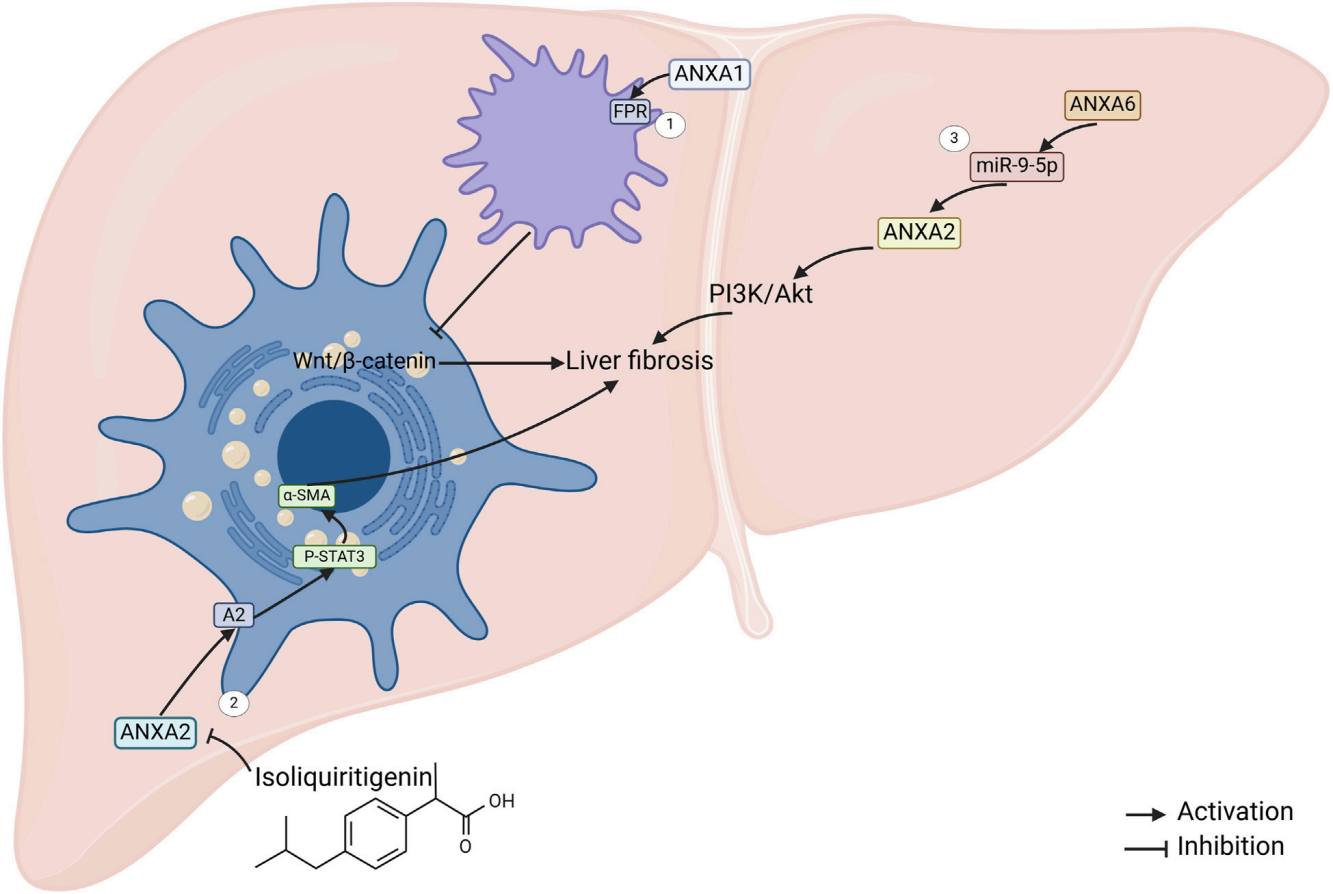
ANXAs involved in liver fibrosis. 1. ANXA1 regulates macrophage activity by targeting formylpeptide receptors, which in turn suppresses the activation of the Wnt/β-catenin pathway in hepatic stellate cells (HSCs), ultimately inhibiting liver fibrosis (Fan et al., 2023). 2. Isoflavones inhibit ANXA2 expression, which leads to a reduction in the phosphorylation of transcription activator 3 (STAT3) and subsequently downregulates the expression of α-SMA (Liu N. et al., 2023). 3. The mouse ANXA6/miR-9-5p/ANXA2 axis, along with the PI3K/Akt pathway, may contribute to liver fibrosis promotion through the long non-coding RNA ANXA2P2 (mouse ANXA6) (Liao et al., 2022).

ANXA2 was significantly upregulated in HBV and alcohol-induced liver fibrosis (Seth et al., 2003; Zhang et al., 2010). ANXA2 levels were significantly elevated in patients with S4 stage of fibrosis compared to those with S0-1 stage of fibrosis (Zhang et al., 2010). Recent research has indicated that isoliquiritigenin suppresses ANXA2 expression. Subsequently, this inhibition reduces the phosphorylation of signal transducer and activator of transcription 3 (STAT3) in downstream signaling pathways (Figure 3). The reduced STAT3 activity downregulates α–smooth muscle actin (α-SMA) expression, ultimately reversing HSC activation and alleviating liver fibrosis (Liu N. et al., 2023). Additionally, the mouse ANXA6/miR-9-5p/ANXA2 axis, along with the PI3K/Akt signaling pathway, may play a role in promoting liver fibrosis mediated by lncRNA ANXA2P2 (Liao et al., 2022). However, whether ANXA2 can be used as a noninvasive biomarker in HBV and alcohol-induced liver fibrosis deserves further investigation.

Furthermore, ANXA3 expression was reduced in liver tissues of mouse models of fibrosis induced by ethanol, olive oil and pyrazole (Jia et al., 2012). In patients with advanced fibrosis associated with HBV infection, ANXA4 expression was significantly increased in liver tissue (Katrinli et al., 2016). These findings suggest that different members of ANXAs may play different roles in the onset and progression of liver fibrosis, and the exact functions of ANXA3 and ANXA4 in liver fibrosis remain unclear warranting further study in the future.

### 3.7 Hepatocellular carcinoma

Liver cancer ranks as the eighth most prevalent cancer and the third leading cause of cancer-related mortality worldwide (Sung et al., 2021). HCC accounts for approximately 80% of all liver cancer cases (Chrysavgis et al., 2022). Although surgery, liver transplantation, chemotherapy, and targeted therapy are the most effective treatment options currently available, the overall survival rate for patients with HCC remains unsatisfactory (Chen et al., 2020). The prognosis is particularly grim for individuals with recurrent disease or distant metastases (Goyal et al., 2013). This highlights the urgent need for further research to develop more effective therapeutic strategies for HCC.

**FIGURE 4:**
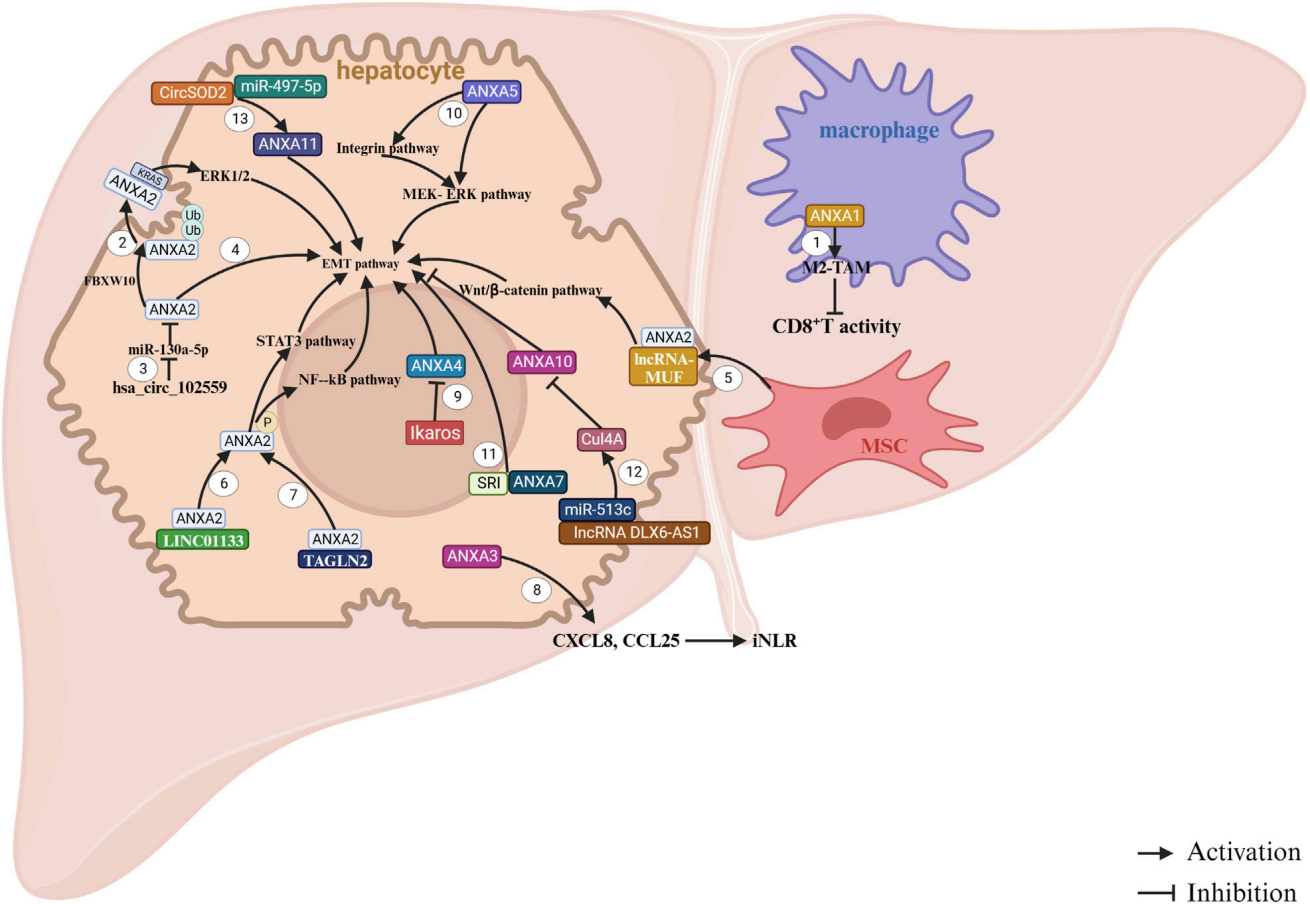
ANXAs involved in HCC. 1. Overexpression of ANXA1 in macrophages promotes malignant growth and metastasis by enhancing the infiltration and M2 polarization of tumor-associated macrophages (TAMs). This process creates an immunosuppressive tumor microenvironment (TME) and suppresses the antitumor response of CD8^+^ T cells (Song et al., 2023b). 2. FBXW10 promotes the polyubiquitination and activation of ANXA2. Once activated, ANXA2 translocates from the cytoplasm to the cell membrane, where it binds KRAS and activates the MEK/ERK pathway, leading to the proliferation of hepatocellular carcinoma (HCC) cells (Liu Z. Y. et al., 2023). 3. The upregulation of hsa_circ_102559 in HCC inhibits the expression of miR-130a-5p, which in turn promotes ANXA2 expression, triggering epithelial–mesenchymal transition (EMT), as well as cell proliferation and migration in HCC (Li et al., 2020). 4. ANXA2 contributes to increased cell proliferation and motility (Shi et al., 2016). 5. LncRNA-MUF binds to ANXA2 and activates Wnt/β-catenin signaling, thereby promoting EMT (Yan et al., 2017). 6. LINC01133 interacts with ANXA2 to activate the ANXA2/STAT3 signaling pathway (Yin et al., 2021). 7. The NF-kB signaling pathway participates in HCC progression through TAGLN2, which interacts with ANXA2 (Shi et al., 2020). 8. ANXA3 enhances the immune response in HCC by inducing the release of chemokines CXCL8 and CCL25, remodeling the immune microenvironment of HCC (Zhu et al., 2020). 9. Overexpression of ANXA4 promotes HCC cell proliferation, but Ikaros can inhibit ANXA4 expression by repressing its promoter activity (Liu et al., 2017). 10. Downregulation of ANXA5 suppresses the expression of molecules in the integrin pathway, such as CRKI/II, DOCK180, and RAC1, as well as key elements in the MEK-ERK pathway (e.g., p-MEK, p-ERK, c-Myc, and MMP-9), along with VIMINTIN in Hca-P cells (Sun et al., 2018). 11. ANXA7 promotes EMT by interacting with SRI, which contributes to the aggressiveness of HCC (Ling et al., 2021). 12. LncRNA DLX6-AS1 increases Cul4A expression by competitively binding to miR-513c. Cul4A then promotes the degradation of ANXA10 through the ubiquitin-proteasome pathway, facilitating the development of HCC (Liu X. et al., 2021). 13. CircSOD2 induces the upregulation of ANXA11 expression by interacting with miR-497-5p (Ye et al., 2023).

In earlier studies, elevated expression of ANXA1 was identified as a predictor of poor prognosis in HCC and was shown to enhance malignant cell behaviors (Lin et al., 2014). Recent studies have revealed that ANXA1 is highly expressed in mesenchymal cells, particularly macrophages, in liver cancer tissues in humans. Furthermore, ANXA1 expression in mesenchymal cells is associated with programmed cell death-ligand 1 (PD-L1) levels. Suppression of ANXA1 expression inhibits HCC cell proliferation and migration by increasing the M1/M2 macrophage ratio and stimulating T-cell activation (Figure 4) (Song et al., 2023b).

Extensive research has highlighted the role of ANXA2 in facilitating the onset and progression of liver cancer. ANXA2 has emerged as a promising prognostic biomarker and modulator of tumor immune microenvironment in various malignancies, including HCC (Ning et al., 2023). Initial findings suggest that ANXA2 is upregulated in human liver cancer tissues and cell lines (Yoon et al., 2006). Subsequent studies have identified ANXA2 as a potential novel marker for tumor angiogenesis in HCC (Yu et al., 2007; Mohammad et al., 2008; Ji et al., 2009; Longerich et al., 2011; Zhang et al., 2012; Sun et al., 2013; Tang et al., 2019; Huang et al., 2021; Herrera-López et al., 2023). Mechanistic investigations have provided further insights. One study has demonstrated that ANXA2 promotes HCC cell migration and invasion *in vitro* by regulating the trafficking of CD147-harboring microvesicles (Zhang W. et al., 2013). In addition, ANXA2 enhances the malignant properties of HCC cells, primarily by remodeling cellular structures (Shi et al., 2016). Additionally, lncRNA-MUF binds to ANXA2, activating the Wnt/β-catenin signaling and epithelial–mesenchymal transition (EMT) (Yan et al., 2017). Furthermore, ANXA2 interacts with engulfment and cell motility protein 1 (ELMO1) to regulate HCC chemotaxis and metastasis (Li et al., 2019). In terms of therapeutic potential, it has been shown that silencing ANXA2 using shRNA effectively reduces hepatoma cell invasion, migration, and tumorigenicity (Zhang H. J. et al., 2013; Dong et al., 2014). Furthermore, ANXA2 is integral in HCC, and its downregulation enhances the efficacy of chemotherapeutic agents such as 5-fluorouracil (Wang et al., 2015). However, serum or tissue ANXA2 levels are not reliable diagnostic markers for HCC in patients with HBV-related cirrhosis and are not associated with patient prognosis.

ANXA3 has been identified as a key factor in conferring resistance to sorafenib in HCC cells, it is enriched in sorafenib-resistant HCC cells and patient-derived xenografts. Mechanistically, ANXA3 overexpression in these cells inhibits the PKCδ/p38-mediated apoptotic pathways while activating autophagy to support cell survival (Tong et al., 2018). Additionally, ANXA3 contributes to chemotherapy resistance in HCC (Pan et al., 2015a). Emerging evidence underscores the pivotal role of ANXA3 in liver cancer stem cell (CSC) maintenance and tumor progression through multiple molecular mechanisms. Studies have demonstrated that both intracellular and secreted ANXA3 significantly enhance the malignant and stem-like properties of CD133^+^ liver CSCs by dysregulating c-Jun N-terminal kinase (JNK) signaling (Tong et al., 2015). Complementary research indicates that ANXA3 sustains HCC CSC activity, potentially through modulation of the hypoxia inducible factor-1A (HIF1A)/Notch signaling axis (Pan et al., 2015b). Additionally, ANXA3 influences chemokine signaling to reshape the infiltrated neutrophil-to-lymphocyte ratio, thereby promoting tumorigenicity in HCC (Zhu et al., 2020). ANXA3 has been identified as an HCC-associated gene, representing a potential therapeutic target for HCC treatment.

The serum level of ANXA4 has been suggested as a potential biomarker for the early detection of HCC (Herrera-López et al., 2023; Saad et al., 2020). Moreover, it has been indicated that reducing ANXA4 expression suppressed HCC cell proliferation and tumorigenesis both *in vitro* and *in vivo* (Liu et al., 2017). Mechanistically, ANXA5 can promote HCC progression and metastasis through the integrin- and mitogen-activated extracellular signal-regulated kinase (MEK)/extracellular regulated protein kinase (ERK) pathway (Sun et al., 2018).

Dysregulation of ANXA7 has been implicated in tumorigenesis, invasion, metastasis, and progression across multiple cancer types, though its functional role appears context-dependent. *In vitro* studies in the human HCC cell line Hep G2 demonstrated that ANXA7 knockdown suppressed cell migration, suggesting its pro-metastatic role in this context (Ibrahim et al., 2013). Similarly, in Hca-F cells—a mouse HCC model with high lymphatic metastatic potential—miR-124-3p exerts tumor-suppressive effects by targeting ANXA7, thereby inhibiting tumor growth, invasion, and lymphatic metastasis (Wang et al., 2020). Mechanistically, ANXA7 interacts with Sorcin (SRI), and their cooperation facilitates EMT, further driving HCC proliferation, invasion, and migration (Ling et al., 2021). However, contrasting findings have been reported. For instance, one study revealed that ANXA7 upregulation suppresses HCC lymph node metastasis, whereas its knockdown exacerbates metastatic spread (Jin et al., 2013).

Unlike other ANXAs, ANXA10 is a tumor suppressor gene (Zhang X. et al., 2023). Elevated ANXA10 expression has been shown to inhibit HCC cell viability, invasion and migration (Liu X. et al., 2021). Conversely, reduced ANXA10 levels in HCC are linked to vascular invasion, early recurrence and poor prognosis, particularly in synergy with p53 mutations (Liu et al., 2002).

### 3.8 Liver regeneration

ANXA6 plays a crucial role in acute liver regeneration. Loss of ANXA6 markedly impairs liver regeneration capacity and reduces survival in mice following partial hepatectomy (PHx) (Enrich et al., 2017). Mechanistic studies revealed that ANXA6 modulates alanine-dependent gluconeogenesis by facilitating the membrane localization and functional recovery of sodium-coupled neutral amino acid transporter 4 (SNAT4). Since alanine is a critical substrate for hepatic gluconeogenesis, ANXA6 deficiency disrupts SNAT4-mediated alanine uptake in hepatocytes, thereby impairing glucose production from alanine. This metabolic disturbance results in paradoxical hepatic alanine underutilization despite elevated plasma alanine levels, ultimately leading to a blockade of the gluconeogenesis pathway and compromised regenerative capacity. Notably, either liver-specific ANXA6 reconstitution or exogenous glucose administration effectively restores normoglycemia and improves survival in PHx mice (Alvarez-Guaita et al., 2020). These findings identify ANXA6 as a key metabolic regulator that orchestrates energy homeostasis during liver regeneration, offering novel insights into the metabolic reprogramming essential for hepatic repair.

## 4 Conclusions and future perspectives

ANXAs represent a family of multifunctional proteins that play significant roles in the pathogenesis of various liver diseases, particularly MAFLD and HCC. This article reviews the progress in research on the functions and mechanisms of ANXAs in liver diseases and offers insights for future research and therapeutic development in this field.

The involvement of ANXA7 in HCC development has been extensively studied; however, its precise function remains controversial. Discrepancies in experimental outcomes may be attributed to several factors: differences in genetic backgrounds, epigenetic modifications, or mutational profiles of HCC cell lines (e.g., Hep G2 vs. Hca-F) across studies could result in divergent ANXA7-associated functions. For example, Hca-F cells exhibit high lymphatic metastatic potential, whereas Hep G2 cells may depend on alternative metastatic pathways. Additionally, due to the complexity of the underlying molecular mechanisms, ANXA7 may exert its effects via different downstream molecules or interacting proteins, the expression or activity of which may vary across cell types. Moreover, variations in gene manipulation techniques (e.g., knockdown vs. knockout), assay conditions, or analytical approaches could also contribute to inconsistent findings. Given the potential dual role of ANXA7 in HCC progression, future research should prioritize the use of standardized model systems and consistent experimental conditions to clarify its function in HCC.

**FIGURE 5:**
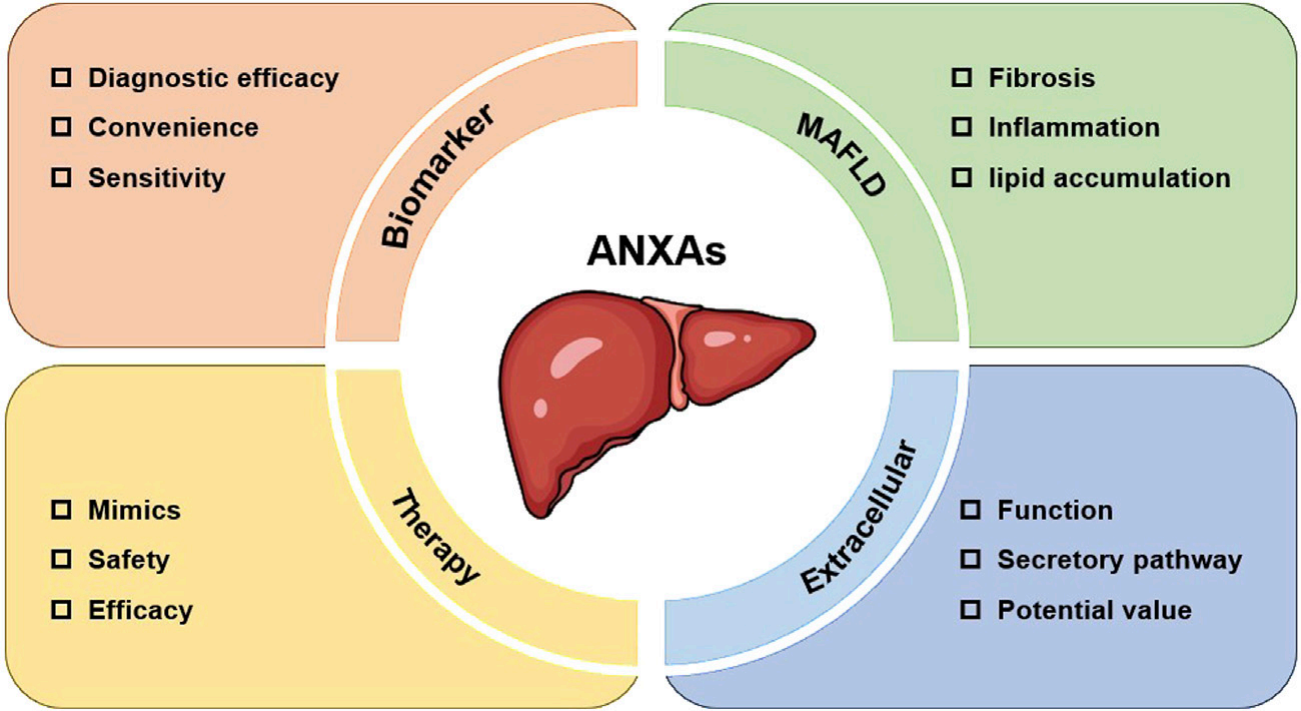
Future perspectives of ANXAs in liver diseases.

The treatment of liver disease is an ongoing area of research, and patient survival rates remains low once progression to end-stage liver disease occurs. Despite a surge of research and findings regarding ANXAs in recent years, gaps still exist in the study of ANXAs and their relationship to liver disease (Figure 5).

Although preliminary studies suggest that ANXAs could serve as promising diagnostic biomarkers for liver diseases, their clinical utility remains uncertain due to the absence of large-scale, multicenter validation studies. Further research is needed to rigorously assess their diagnostic sensitivity and specificity, ideally through comprehensive comparisons with established liver disease biomarkers.

MAFLD has now become the leading cause of chronic liver disease. Although the epidemiology of MAFLD has been extensively studied, the underlying mechanisms and effective therapeutic targets remain unclear. Members of ANXAs, such as ANXA1 and ANXA5, have demonstrated significant improvement of inflammation and antifibrotic effects in MAFLD. However, there are some ANXAs with unknown roles in MAFLD. Considering the similarities among members of the same family, we hypothesize that other ANXAs also play important roles in MAFLD, but the specific roles and mechanisms require further investigation in the future.

Notably, while the secretion of certain ANXAs into the extracellular environment has been reported, the precise mechanisms governing their secretion and their functional consequences in the extracellular milieu remain poorly characterized. Moreover, although several ANXAs have established biological roles, the molecular mechanisms mediating these functions are incompletely understood.

An increasing body of evidence highlights the therapeutic potential of targeting ANXAs for liver diseases treatment. For instance, ANXA6 administration significantly enhances survival in PHx mouse models, suggesting its promise as a regeneration-promoting drug for the treatment of acute liver injury and even liver failure. Additionally, ANXA5 has demonstrated strong ability to improve lipid metabolism, inflammation and fibrosis in MAFLD mice. The development of mimics or neutralizing antibodies against ANXAs with great therapeutic potential for clinical use would bring new hope to patients with liver diseases.

In summary, ANXAs hold great promise as biomarkers and therapeutic targets for liver diseases. Understanding their nuanced roles in disease-specific contexts will be crucial for translating these insights into effective and targeted therapies.
