# Supplementary material for: Role of the Annexin A protein family in liver diseases: insights and therapeutic opportunities
Source: Front Pharmacol. 2025 Jul 11;16:1569927. doi: 10.3389/fphar.2025.1569927 (PMC12289494; doi:10.3389/fphar.2025.1569927)
Supplement: Supplementary file 1 [file Table1.docx]

**Supplementary Table 1. Characteristics and expression of Annexin A proteins.**

| Protein | Alias | chromosome localization | molecular weight, kDa | expression |
| --- | --- | --- | --- | --- |
| ANXA1 | ANX1, LPC1 | 9q21.13 | 34 kDa | esophagus, bone marrow, placenta, fat, lung, gall bladder |
| ANXA2 | ANX2, ANX2L4, LPC2, LPC2D, P36, CAL1H, HEL-S-270, LIP2, PAP-IV | 15q22.2 | 38 kDa | Ubiquitous expression in various tissues |
| ANXA3 | ANX3 | 4q21.21 | 33 kDa | Broad expression in various tissues |
| ANXA4 | ANX4, PIG28, PP4-X, HEL-S-274, P32.5, PAP-II, ZAP36 | 2p13.3 | 40.8 kDa | Broad expression in various tissues |
| ANXA5 | ANX5, PP4, CPB-I, ENX2, HEL-S-7, RPRGL3, VAC-alph | 4q27 | 36.75 kDa | Ubiquitous expression in various tissues |
| ANXA6 | ANX6, p70, , CBP68, CPB-II, p68 | 5q33.1 | 60-90 kDa | Ubiquitous expression in various tissues |
| ANXA7 | ANX7, SYNEXIN, SNX | 10q22.2 | 47 kDa | Ubiquitous expression in various tissues |
| ANXA8 | ANX8, CH17-360D5.2 | 10q11.22 | 36 kDa | skin, esophagus, urinary bladder, placenta, lung |
| ANXA9 | ANX31 | 1q21.3 | 38 kDa | Broad expression in various tissues |
| ANXA10 | ANX14 | 4q32.3 | 28-35 kDa | stomach, urinary bladder, liver |
| ANXA11 | ALS23, ANX11, CAP-50, CAP50, I | 10q22.3 | 56 kDa | Ubiquitous expression in various tissues |
| ANXA13 | ANX13, ISA | 8q24.13 | 33 kDa | duodenum, small intestine, colon, liver, kidney |
